# Elmira Surgical Institute

**Published:** 1873-10

**Authors:** 


The Bistoury.

ELMIRA, N. Y., OCT. 1873.

The BISTOURY is published Quarterly, upon the 1st of April, July, October and January, at Fifty Cents a year, in advance.

### The Elmira Surgical Institute.

By the time this number of the BISTOURY reaches our readers, a new Institution, the cherished scheme of our life, will have sprung into existence. A twelve years arduous private practice has confirmed the opinion, long since formed, that an Institution, similar to the one we are engaged in building, is sadly needed and will be gladly welcomed by those who are unable to pay the enormous expense entailed by a lengthy stay in New York or Philadelphia, the only cities where orthopaedic hospitals are afforded. We have purchased a large building, beautifully located and convenient of access, which we are fitting up especially with a view of affording good board and comfortable beds, at prices within the reach of those of the most limited circumstances, who require surgical treatment for the deformities of body and limb, and for the cure of blindness.

In this Institution, we will be able to accommodate all classes and conditions of society, with apartments suited to their tastes and means, and will not be forced to subject them to the inconvenience of seeking boarding places about the city, as formerly. By the first of January at latest, we will have the Institution open and our private offices re moved to the building, where we will be ready to welcome our friends, and exhibit to them a perfect model of a Surgical Institute.

